# Long-Term Oncologic Outcomes of Image-Guided Irreversible Electroporation for Localized Prostate Cancer

**DOI:** 10.1007/s00270-024-03826-6

**Published:** 2024-08-07

**Authors:** Federico Collettini, Carsten Stephan, Thomas Fischer, Andreas Maxeiner, Timo Alexander Auer, Bernhard Gebauer

**Affiliations:** 1 Department of Radiology, Charité - Universitätsmedizin Berlin, Corporate Member of Freie Universität Berlin, Humboldt-Universität zu Berlin, and Berlin Institute of Health, Charité Campus Mitte (CCM), Charitéplatz 1, 10117 Berlin, Germany; 2 Department of Urology, Charité - Universitätsmedizin Berlin, Corporate Member of Freie Universität Berlin, Humboldt-Universität zu Berlin, and Berlin Institute of Health, Charité Campus Mitte (CCM), Charitéplatz 1, 10117 Berlin, Germany; 3 Berlin Institute of Health (BIH), Anna-Louisa-Karsch-Straße 2, 10178 Berlin, Germany; 4 Berlin Institute for Urologic Research, Berlin, Germany

Editor,

Despite excellent functional results and good short-term oncologic outcomes, the acceptance of focal therapies in the treatment of localized prostate cancer (PCa) is forcibly limited by the lack of mid- and long-term oncologic outcomes [1, 2]. This is particularly true for focal PCa ablation using Irreversible Electroporation (IRE), which—because of its non-thermal mode of action and assumed relative sparing of nerve structures—has attracted considerable attention from both physicians and patients. The Prostate cancer: outcome with irreversible Electroporation of localized tumors trial was a single-center prospective study that has shown promising midterm results, both in terms of urogenital toxicity and oncologic control in patients with localized, low- to intermediate-risk PCa treated with MRI–transrectal US fusion–guided IRE [3]. Here, we present the 5-year oncologic outcomes of the POTENT Trial.

Study design, baseline data and midterm functional and oncologic outcomes of the study have been previously reported [3]. Thirty consecutive men with localized, low- to intermediate-risk PCa (PSA level, ≤ 15 ng/mL; Gleason score, ≤ 3 + 4; clinical stage, ≤ T2c; lesion size at multiparametric prostate MRI, ≤ 20 mm) underwent focal MRI–transrectal US fusion–guided IRE of the prostate. The study had been approved by the responsible ethic committee (EA4/052/13). 5-year oncologic outcomes to be reported include: (1) failure-free survival (FFS), defined as avoidance of local salvage radical therapy (SRT), systemic therapy, metastases, and prostate cancer–specific death; (2) metastasis-free survival; (3) prostate cancer–specific and overall mortality and (4) time to radical therapy (TTRT), defined as the time from the first IRE treatment to the time point of radical therapy (surgery or radiotherapy). Statistical analysis was conducted using the R statistics package (version 3.4.2; R Foundation, Vienna, Austria). Discrete and continuous variables are displayed as median and interquartile range (IQR), whereas categorical variables are displayed as frequencies and percentages.

The overall median follow-up was 64.5 months (IQR 44.7–69 months) and it was 68 months (IQR 65.2–76.7 months) for participants with no reported events (n = 19). During the 5-year of follow-up, 7/30 participants (23%) transitioned to salvage radical prostatectomy and 4/30 participants (13%) to salvage external beam radiotherapy (Fig. 1). FFS rate was 97% at 1 year, 87% at 2 years, 77% at 3 years, 70% at 4 years and 63% at 5 years (Fig. 2). For participants that transitioned to SRT the median TTRT was 32 moths (IQR 22.5–45.5 months). Transition to SRT was due to non-significant cancer in 6/11 (55%) and significant cancer in 5/11 (45%) participants at follow-up biopsy. 5-year metastasis-free survival, prostate cancer–specific survival and overall survival were 100%.

**Fig. 1:**
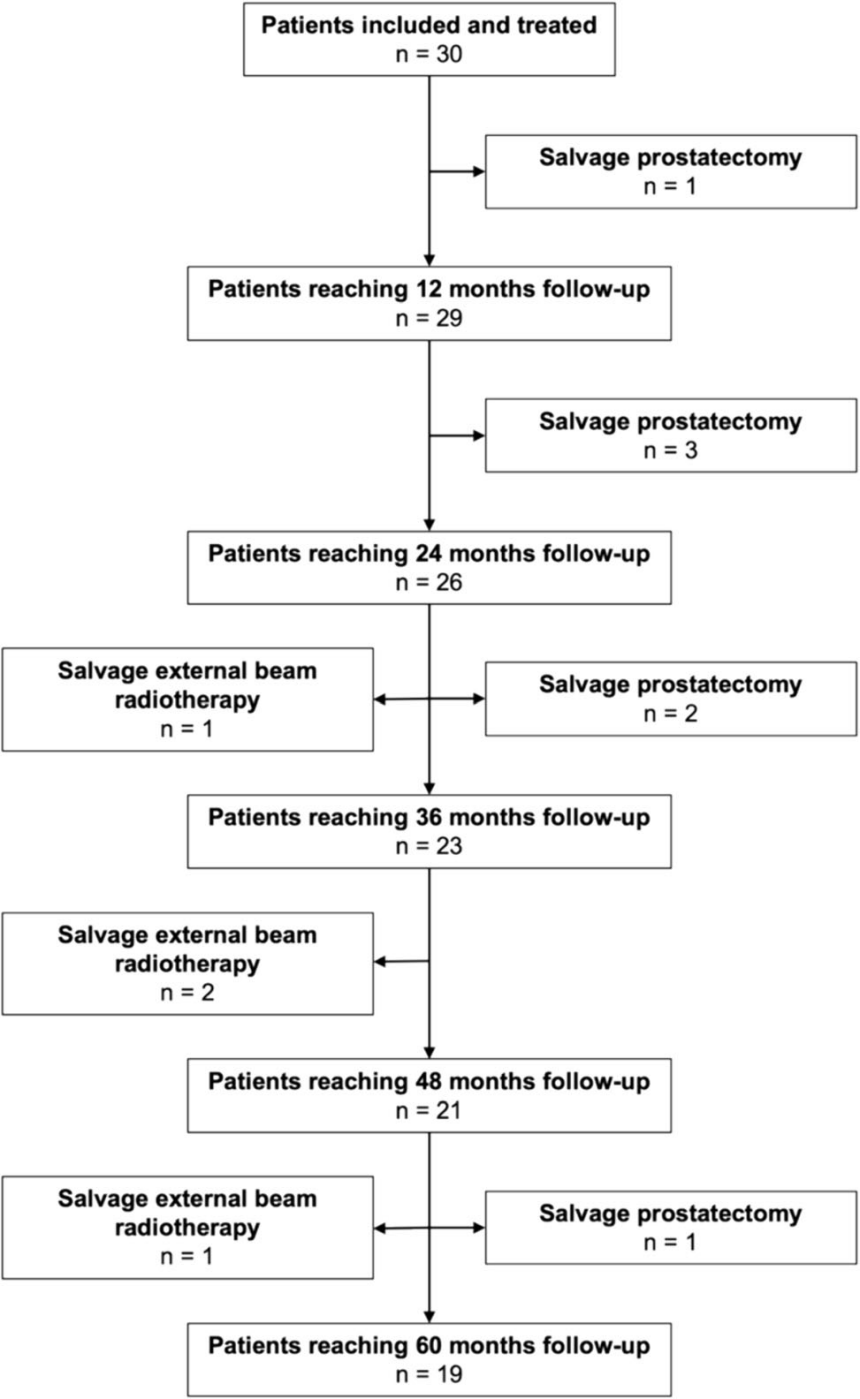
Study flowchart

**Fig. 2:**
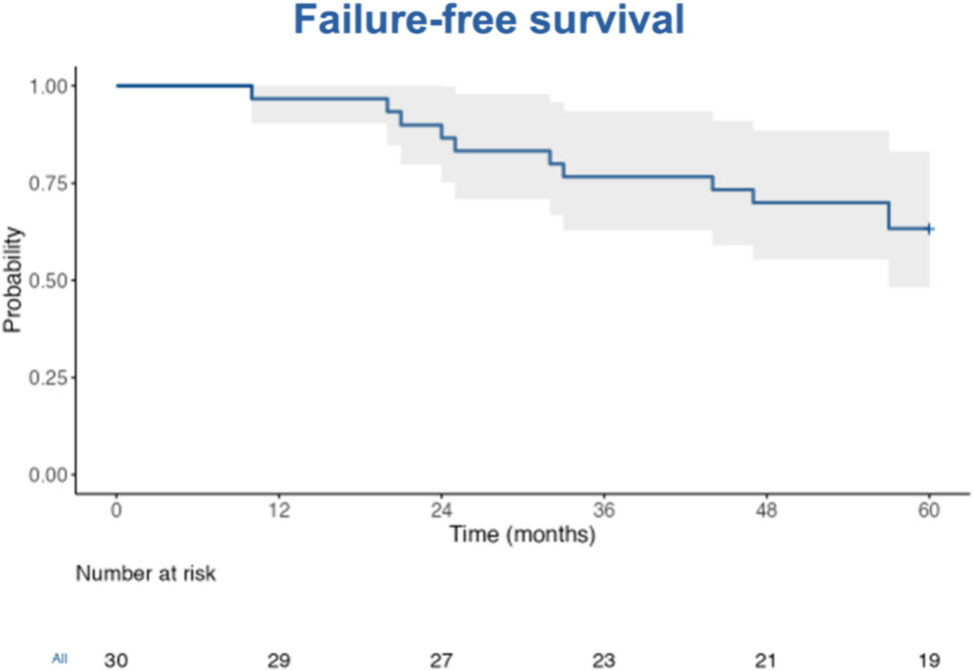
Failure-free survival

In our cohort with ~ 3/4 of intermediate-risk PCa, FFS rate after focal IRE was 63% at 5 years. Median TTRT was 32 months. 5-year metastasis-free survival, prostate cancer–specific survival and overall survival were 100%. The FFS rates reported herein are less favorable than reported 5-year outcomes following focal cryoablation and HIFU (75% and 82% FFS at 5 years, respectively) [4]. This is most probably attributable to the low threshold for progression to salvage treatment adopted by the treating urologists. In fact, in most patients (55%) who underwent radical therapy during follow-up, transition to SRT was due to non-significant cancer detected at prostate biopsy during follow-up. Focal PCa therapies are still not widely recognized among practicing urologists, and although recommendations for the management of recurrent or persistent disease after focal therapy exist, they are frequently not implemented in real-world scenarios [5]. In conclusion the long-term oncologic results of our study shown that for selected patients who are open to taking on the potential risk of needing salvage radical therapy in exchange for maintaining baseline urinary and sexual function, focal IRE may be a safe and effective minimally-invasive treatment alternative.

## References

[1] EAU Guidelines. Edn. presented at the EAU Annual Congress Milan 2023. ISBN 978-94-92671-19-6.

[2] Eastham JA, et al. Clinically localized prostate cancer: AUA/ASTRO guideline, part II: principles of active surveillance, principles of surgery, and follow-up. J Urol. 2022;208(1):19–25. 35536148 10.1097/JU.0000000000002758

[3] Collettini F, et al. Image-guided irreversible electroporation of localized prostate cancer: functional and oncologic outcomes. Radiology. 2019;292(1):250–7. 31161973 10.1148/radiol.2019181987

[4] Faiella E, et al. Focal minimally invasive treatment in localized prostate cancer: comprehensive review of different possible strategies. Cancers. 2024;16(4):765. 38398156 10.3390/cancers16040765 PMC10887212

[5] Tay KJ, et al. Surveillance after prostate focal therapy. World J Urol. 2019;37(3):397–407. 29948045 10.1007/s00345-018-2363-y

